# Banana Peel (*Musa* ABB cv. Nam Wa Mali-Ong) as a Source of Value-Adding Components and the Functional Properties of Its Bioactive Ingredients

**DOI:** 10.3390/plants13050593

**Published:** 2024-02-22

**Authors:** Pattarapol Khamsaw, Sarana Rose Sommano, Malaiporn Wongkaew, William G. T. Willats, Cassie R. Bakshani, Sasithorn Sirilun, Piyachat Sunanta

**Affiliations:** 1 Plant Bioactive Compound Laboratory, Faculty of Agriculture, Chiang Mai University, Chiang Mai 50200, Thailand; pattarapol_kha@cmu.ac.th (P.K.); sarana.s@cmu.ac.th (S.R.S.); malaiporn@rmutl.ac.th (M.W.); 2 Department of Pharmaceutical Sciences, Faculty of Pharmacy, Chiang Mai University, Chiang Mai 50200, Thailand; 3 Department of Plant and Soil Sciences, Faculty of Agriculture, Chiang Mai University, Chiang Mai 50200, Thailand; 4 Program in Food Production and Innovation, College of Integrated Science and Technology, Rajamangala University of Technology Lanna, Chiang Mai 50220, Thailand; 5 Department of Biology, School of Natural and Environmental Sciences, Newcastle University, Tyne NE1 7RU, UK; william.willats@newcastle.ac.uk (W.G.T.W.); c.r.bakshani@bham.ac.uk (C.R.B.); 6 Institute of Microbiology and Infection, College of Medical and Dental Sciences, University of Birmingham, Birmingham B15 2SQ, UK; 7 Innovation Center for Holistic Health, Nutraceuticals and Cosmeceuticals, Faculty of Pharmacy, Chiang Mai University, Chiang Mai 50200, Thailand; 8 Research Unit for Innovation in Responsible Food Production for Consumption of the Future (RIFF), Multidisciplinary Research Institute, Chiang Mai University, Chiang Mai 50200, Thailand

**Keywords:** antioxidant properties, banana by-product, glycoarray, prebiotic activity, prebiotic index

## Abstract

Banana peel (BP) is the primary by-product generated during banana processing which causes numerous environmental issues. This study examines the physical attributes, proximate analysis, glycoarray profiling, antioxidant abilities, and prebiotic activity of BP. The analysis demonstrated that carbohydrates constituted the primary components of BP and the glycoarray profiling indicated that BP contains multiple pectin and hemicellulose structures. BP also contained phenolic compounds, including (+)-catechin and gallic acid, flavonoid compounds, and antioxidant activities. BP demonstrated prebiotic effects by promoting the proliferation of advantageous gut bacteria while inhibiting the growth of harmful bacteria. The prebiotic index scores demonstrated that BP exhibited a greater capacity to promote the growth of beneficial bacteria in comparison to regular sugar. The study demonstrated the potential of the BP as a valuable source of dietary fibre, bioactive compounds, and prebiotics. These components have beneficial characteristics and can be utilised in the production of food, feed additives, and functional food.

## 1. Introduction

Bananas are popular tropical fruits renowned for their high acceptance, wide availability, affordability, and abundant supply that plays a vital role in ensuring food security. Bananas have also become a dessert fruit and a staple starch crop in numerous regions, highlighting their significant dietary value [1,2]. In 2022, the global banana production reached 125 million tons [3,4]. Bananas are not only sold as fresh fruits but are also utilised in various processed products [5,6]. However, the amount of biomass generated during banana preparation is enormous; in fact, the peel accounts for 35–40% of the total weight [7]. In Thailand, ‘Nam wa mali-ong’ bananas are an important variety due to their desirable characteristics and suitability for various food manufacturing processes. The annual productivity of this banana in the northern region alone is approximately 89,000 tons, resulting in up to 35,000 tons of peel biomass [8]. Typically, the biomass is buried or repurposed for fertilisation. 

Banana peel (BP) is abundant in dietary fibre and rich in bioactive compounds, making it a valuable resource [9,10,11]. Some of these natural components exhibit remarkable antioxidant, antibacterial, and antibiotic properties, making BP a promising candidate for nutraceutical and pharmaceutical applications [12,13,14]. The peel has historically served as a medicinal treatment for different illnesses, including burns, anaemia, gastrointestinal disorders, ulcers, inflammation, diabetes, respiratory diseases, and abnormal uterine bleeding [15]. Interestingly, BP contains a high concentration of dietary fibre and phenolic compounds, further enhancing its potential health benefits, including antioxidant capacity, antimicrobial, antibiotic, and prebiotic properties [16,17,18]. Remarkably, the total phenolic content in BP is 1.5–3 times greater than the quantity found in the pulp [19]. Therefore, it holds great potential for future utilisation in food and feed additives, dietary supplements, and pharmaceutical products. Through the extraction and utilisation of multiple valuable components from biomass, biorefinery processes can enhance resource efficiency, minimise waste, and promote sustainable practices [20,21,22].

To date, there have been limited analyses of the valorisation potential of BP, including specifically ‘Nam wa mali-ong’ bananas. This study aims to address this knowledge gap by investigating the biomass volume generated during processing and identifying the valuable components present in BP. Additionally, the study evaluated the value-adding compositions in the alcoholic fraction and tested its antioxidant properties and prebiotic potential, specifically focusing on their interaction with human intestinal probiotics. This work sheds light on the untapped potential of ‘Nam wa mali-ong’ BP in biorefinery applications and highlights the potential of the value-adding components from BP for functional food development and in promoting the sustainable utilisation of this important agricultural by-product.

## 2. Results and Discussion

### 2.1. Banana Peels’ Preparation and Processing Flow Diagram 

**Figure 1:**
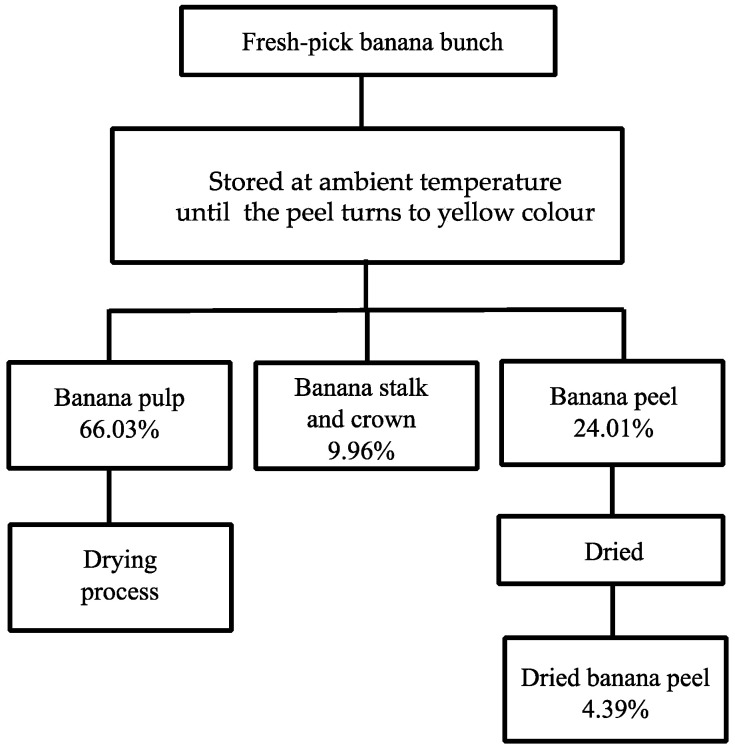
The processing flow diagram coup with mass flow balance of dried banana manufacturer.

The processing flow diagram of the pre-processing of bananas in a dried banana manufacturing company is shown in Figure 1. The composition of the ripe banana branch is comprised of 9.69% banana stalk, 24.01% banana peel, and 66.03% banana pulp. The BP is the main by-product of dried banana processing, which is generated at a rate of more than 700 kg per day, only by this manufacturer. However, the moisture content of banana skin exceeds 95%.

**Figure 2:**
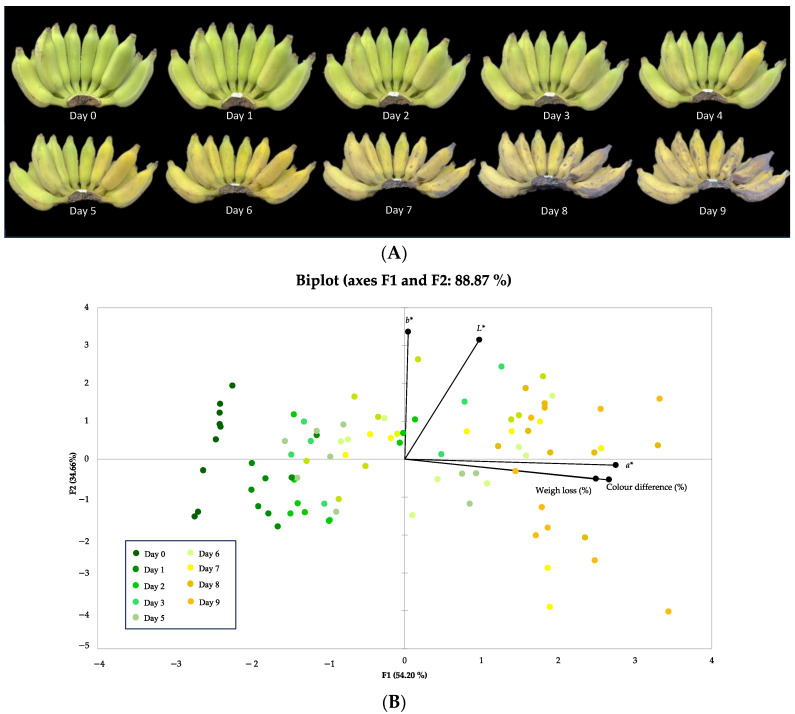
(**A**) The appearance of bananas during the storage. (**B**) The PCA analysis of bananas’ physical properties during storage.

The changes in colour and weight loss of bananas during storage are presented in Table 1. The L* value, which represents lightness, remained unchanged during the storage period; however, the a* value, which shows the green- and red-coloured components, demonstrated an increase over time. On the day of harvest, the banana peel had a green colour with an a* value of approximately −8.78. In a period of 9 days of storage, the colour gradually changed to yellow, corresponding to an increase in the a* value to 13.89. These data suggest that the green colour declined considerably over the duration of storage, as presented in Figure 2A. However, the observed variable b* did not show a statistically significant difference over the period of storage. The variable ∆E* refers to the measure of colour alteration in the peel of a freshly harvested banana. It is evident that the colour of the banana peel has significant changes over the period of storage. According to X-t Yang et al. [23], the change in peel colour is contingent upon the degradation of chlorophyll. However, it is also seen that the rate of chlorophyll degradation is influenced by the storage temperature. Furthermore, the overall weight loss was shown to be significantly reduced over the period of storage because of the moisture loss. Typically, the manufacturer would utilise bananas that have been stored for 6 days, exhibiting a mostly yellow colour and devoid of any brown spots, for the purpose of banana processing.

A principal component analysis (PCA) model was applied in order to present the whole dataset using a plot that has reduced dimensionality. The correlation between the duration time of storage and the physical characteristics of bananas is depicted in Figure 2B, which accounts for 88.87% of the observed variability as indicated by the biplot. The PCA revealed a positive correlation between the a* value, weight reduction (%), and ∆E* or colour difference. Nevertheless, this model states that there is a correlation between the duration of storage and the a* value, weight loss percentage, and ∆E* value.

In this manufacturer, the bananas were subsequently subjected to a peeling process, followed by drying in a parabolic dome. It was then subjected to a drying process in a hot-air oven and packed using a vacuum-sealer. 

### 2.2. Proximate Analysis

The result of proximate analysis is shown in Table 2. The major component of BP were carbohydrates and fibres, which accounted for 61.34% and 16.56%, respectively. According to Emaga et al. [24], the primary components identified in BP included dietary fibre (43–50%), fat (3–10%), protein (6–9%), and starch (3%). The nutrients in BP are dependent upon various factors, including its variety and stage of ripeness. Furthermore, it should be noted that the BP contains a significant amount of dietary fibre, including up to 20% of pectin. This particular composition made it potentially valuable in both the food and feed additive industries.

### 2.3. Glycoarray Profiling

Microarray polymer profiling (MAPP) has proven to be an effective technique for elucidating the relative abundance of multiple glycans in plant materials [25]. MAPP involves the sequential extraction of glycans from homogenised samples which are then printed as high-density microarrays and subsequently probed with antibodies or carbohydrate-binding modules with known specificities against carbohydrate structures. 

H_2_O is often used for initial extraction to release highly soluble polysaccharides. This may be followed by cyclohexanediamine tetraacetic acid (CDTA), NaOH, and cellulase [26]. CDTA, a chelating agent, disrupts calcium ions that cross-link pectin molecules in the cell wall, allowing the pectic polysaccharides to be solubilised and extracted. NaOH disrupts hydrogen bonds and ester linkages present in the cell wall matrix, leading to the solubilisation of hemicelluloses and associated polymers. Cellulase degrades, releasing smaller glycan chains and other glycans that may be entrapped in, or associated with the microfibrils.

**Figure 3:**
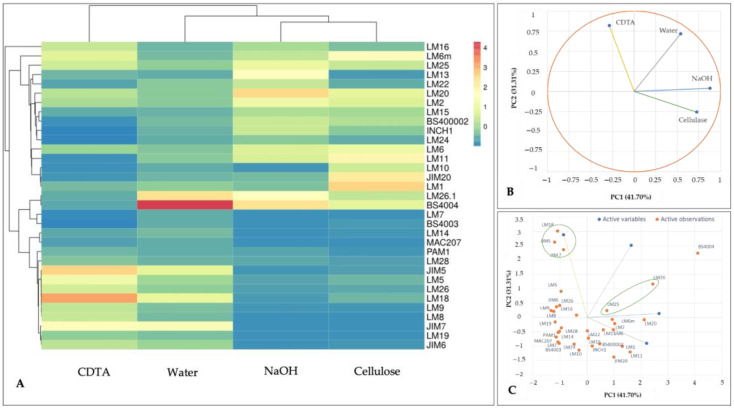
Heatmap diagram of glycoactive ingredients recovered by CDTA, NaOH, H_2_O, and cellulase reagents (**A**); loading plot (**B**) and biplot (**C**) of the monoclonal antibodies in comprehensive microarray polymer profiling. The green circle in Figure 3C symbolises the potential correlation between the glycoactive ingredients and the antibody.

MAPP data are presented as a heatmap in Figure 3A and a list of the antibodies used and their specificities is shown in Table 5. As expected, H_2_O and CDTA released a wide range of more soluble polysaccharides, including many pectins such as arabinans (recognised by LM6 and LM6-M), galacan (recognised by LM5 and LM26), and homogalacturonan (recognised by JIM5, JIM7, LM18, LM19, PAM1, and LM7). Arabinogalactan protein epitopes were also detected in these fractions, for example, recognised by LM14, LM16, and LM2. Interestingly, the mannan epitope recognised by BS-400-4 was also highly abundant in the H_2_O fraction. Also, as expected, the NaOH and cellulase fractions were mostly dominated by hemicellulose epitopes that require harsher solvents to release from cell wall materials. This included xyloglucan (recognised my LM15 and LM25), manna, recognised by LM22xylna (recognised by LM10 and LM11) and (1-3)-beta-glucan (recognised by BS-400-2). Taken together, these data indicate that BP is comprised of a rich variety of polysaccharides and glycoproteins. 

To understand the correlation of the monoclonal antibodies and extracts, a thorough analysis was performed to determine the principal component analysis (PCA). The loading plot (Figure 3B) shows that 41.7% of the data were depicted in PC1 and 31.31% were in PC2. Similarly, different types of glycosides were derived by these solvents, in which the extracts with CDTA projected separately from the others. The biplot depicted in Figure 3C provides insights that this glycoside extract was recognised by LM18, JIM5, and JIM7 antibodies. In the context of pectin structural analysis, LM18 was specifically employed to identify (1-4)-β-D-galactan [27], while JIM5 and JIM7 picked up partially low methyl-esterified HG epitope [28]. LM25 and LM26 played a significant role in the glycan extracts treated with H_2_O, NaOH, and cellulase. These antibodies identified epitopes associated with β-1,6-galactosyl substitution on β-1,4-galactan and galactosylated xyloglucan. This recognition suggests that these polysaccharides contain β-glucan epitopes [29,30,31]. NaOH and cellulase treatments predominantly extracted polysaccharides likely recognised by LM6 and LM2 antibodies. This suggests the presence of (1→5)-α-L-arabinan and Arabinogalactan protein (AGP) structures featuring β-linked glycosylglucopyranuronic acid (GlcA) [32,33,34]. Cellulose is a linear polysaccharide composed of β-1,4-linked glucose units. The presence of (1→5)-α-L-arabinan indicates a component of hemicellulose with arabinose side chains, suggesting a specific hemicellulosic structure. From our data, we want to highlight that the fractionation process using NaOH successfully retrieved methyl-esterified pectic homogalacturonan, as indicated by its recognition by the LM20 antibody [35]. 

### 2.4. The Antioxidant Propeties 

Table 3 provides a comprehensive overview of the antioxidant characteristics exhibited from the BP. The BP extracts obtained by methanol and dichloromethane extraction exhibited a total phenolic content of 30.71 ± 3.15 and 36.23 ± 1.87 mg gallic/g dry weight, respectively. Additionally, the extracts showed that the total flavonoid content of 12.82 ± 2.05 and 27.99 ± 2.01 mg (+)-catechin/g dry weight, respectively. These findings suggest that the choice of solvents has an impact on the solubility of the active ingredients in the BP powder. The extracts obtained through methanol extraction exhibited lower levels of total phenolics and total flavonoid contents compared to those obtained through dichloromethane extraction. 

In another research, the extraction of BP was conducted using several solvents, and the subsequent analysis was performed using a specific methodology [36]. The composition and polyphenol concentration of bananas were influenced by the extraction procedure and the types of solvents used. For instance, methanol exhibits greater polarity compared to dichloromethane, enabling it to extract a larger quantity of phenolics and flavonoids [37]. Furthermore, the isolated material has superior antioxidative properties. To achieve this, the extraction process involves the initial use of dichloromethane followed by methanol. Furthermore, apart from extracting extremely valuable compounds, it also enhances the extraction efficiency significantly compared to the utilisation of a single solvent. Based on the existing findings, it has been determined that BP of Kluai Nam Wa Mali Ong serves as a genuine reservoir of polyphenols. Regarding the assessment of antioxidant radical scavenging activity from BP, three methods have been employed: 2,2’-azino-bis (3-ethylbenzthiazoline-6-sulphonic acid) or ABTS, 2,2-diphenyl-1-picrylhydrazyl test or DPPH, and the ferric reducing antioxidant power or FRAP. The ABTS value of banana peel extracts obtained by the use of methanol and dichloromethane solvents were 34.36 ± 6.22 and 15.31 ± 0.43 mg trolox/g sample, respectively; the DPPH values of BP extracts obtained using methanol and dichloromethane were determined to be 52.38 ± 2.04 and 42.66 ± 1.75 mg trolox/g sample, respectively. Additionally, the FRAP values of the banana peel extract obtained using methanol and dichloromethane were found to be 0.36 ± 0.01 and 0.15 ± 0.01 mg ascorbic acid/g sample powder, respectively. It is noteworthy that the antioxidant values obtained from banana peels extracted with methanol were higher than those obtained from banana peels extracted with dichloromethane using all three methods. The HPLC method was employed to examine the phenolic components recovered from BP. The analysis revealed that the methanol solvent extraction yielded two distinct types of phenolic compounds, (+)-catechin and gallic acid, with corresponding yields of 0.24 and 0.22 mg/g dry weight. Regarding the chemicals obtained through extraction using the solvent dichloromethane, two distinct types of phenolic substances, labelled as *p*-coumaric acid and rosmarinic acid, were acquired. The yields for these substances were determined to be 0.58 and 0.93 mg/g dry weight, respectively.

According to Wongwaiwech [38], the chemical properties of the ‘Nam Wa Mali-Ong’ banana pulp exhibited lower levels of total phenolic content (229 mg gallic acid equivalent/100 g dry weight), total flavonoid content (89 mg rutin equivalent/100 g dry weight), and DPPH (437 mg trolox equivalent/100 g dry weight), but higher levels of FRAP (961 mg FeSO_4_ equivalent/100 g dry weight). This disparity can be attributed to variations in the standard equivalent used for analysis. Consistent with the findings of Tongkaew et al. [39], this study discovered the presence of (+)-catechin and gallic acid in fresh banana peel, with concentrations of 4.59 mg/g fresh weight and 0.24 mg/g fresh weight, respectively. These compounds were evaluated using RP-HPLC-DAD and was found to have greater levels. In line with Wongwaiwech et al. [38], the levels of (+)-catechin and gallic acid in fresh banana pulp were measured using HPLC-DAD and MS. The results showed that the concentration of (+)-catechin was 0.10 mg/g fresh weight, while the concentration of gallic acid was 0.03 mg/g fresh weight. Oliveira et al. [40] reported the identification of trace amounts of *p*-coumaric acid (<0.005 mg/g dry weight) in banana peels.

Plant antioxidants are the phytochemicals that can neutralise free radicals, the molecules with free electrons. These molecules have the potential to disrupt cellular components such as proteins, lipids, and DNA via oxidative stress. Moreover, it is believed that oxidative stress plays a role in the ageing process and the pathogenesis of chronic diseases such as cancer, cardiovascular disease, and neurological disorders [11]. Plant antioxidants demonstrate the ability to donate electrons to free radicals, thereby stabilising them and mitigating their deleterious consequences. Bananas are recognised as a valuable source of antioxidants, with significant amounts of vitamin C, vitamin A, and polyphenol compounds. Nevertheless, the antioxidant composition of a banana may display variability based on factors such as the level of ripeness and their variety [41]. It is noteworthy that the peel of the banana contains a greater abundance of phenolic compounds compared to the flesh of the fruit. The composition of banana peel consists of around 40 distinct compounds, including hydroxycinnamic acids, flavonols, flavan-3-ols, and catecholamines [15]. The primary flavonoids found in banana peels are rutin and its conjugates, while the main hydroxycinnamic acid present is ferulic acid. Moreover, flavan-3-ols, including proanthocyanidins, constitute a majority of phenolic compounds found in BP. BP contains significant amounts of dopamine and L-dopa, which are responsible for the antioxidant qualities exhibited by the peel. Similar to banana flesh, the quantity of antioxidants is dependent on many factors, including variety, production conditions, and fruit age [42]. Moreover, the method of recovery is also influential to the quantity of the antioxidants. For instance, heat treatments can release bound bioactive molecules and enhance antioxidant activity, while as mentioned, the polarity of methanol also plays a role. 

### 2.5. Prebiotic Activity

Observations have been collected on the growth of four diverse microorganisms comprising two advantageous strains, namely *Lacticaseibacillus paracasei* and *Bifidobacterium longum*, and two harmful strains, especially *Escherichia coli* and *Clostridium perfringens*. The analysis of microorganisms revealed that the groups given BP extract and inulin displayed a higher rate of bacterial growth, with values of 9.96 ± 0.09 and 9.96 ± 0.06 Log_10_ cfu/mL, respectively, compared to regular sugar (9.41 ± 0.22 Log_10_ cfu/mL). The population of beneficial bacteria in the media increased significantly when using BP extract (9.59 ± 0.06; 9.41 ± 0.04 Log_10_ cfu/mL for *L. paracasei* and *B. longum*, respectively) and inulin (9.58 ± 0.10; 9.23 ± 0.06 Log_10_ cfu/mL for *L. paracasei* and *B. longum*, respectively), compared to using glucose (8.72± 0.10; 7.63 ± 0.10 Log_10_ cfu/mL for *L. paracasei* and *B. longum*, respectively) as a carbon source. However, the growth of *E. coli* and *C. perfringens* was slower when given inulin (7.82 ± 0.13; 6.56 ± 0.13 Log_10_ cfu/mL) compared to glucose (8.06 ± 0.15; 7.40 ± 0.10 Log_10_ cfu/mL) and BP extract (8.14 ± 0.11; 7.47 ± 0.13 Log_10_ cfu/mL).

The prebiotic index evaluates the capacity of a particular substrate to promote the growth of a beneficial microorganism relative to other microbes, as well as the growth on a general carbon source, such as glucose or any other sugar used as a control. The bacterial prebiotic index values in Table 4 show a substantial increase when supplemented with BP extract (1.64 ± 0.09) and inulin (1.77 ± 0.07), compared to glucose (0.43 ± 0.07), and have a numerical value of greater than 1. The use of BP extract has the capacity to enhance the proliferation of advantageous microorganisms similar to inulin, while inulin is a prebiotic that has the ability to enhance the growth and activity of probiotics and is frequently employed as a component in the functional food industry. According to the research conducted by Kaewarsar et al. [43], the PI score of inulin in co-culture after 48 h was notably greater than that of the control (glucose) and above 1, suggesting its efficacy in promoting the growth of probiotic strains. Nevertheless, the peel of bananas contains abundant nutrients that are useful for *Lactobacillus* spp., a type of bacteria that has a crucial function in inhibiting the proliferation of harmful microorganisms. Probiotic bacteria have actions such as adjusting pH, competing for food, and producing antimicrobial chemicals to prevent the growth of harmful microorganisms [44]. According to Zahid et al. [45], there were no notable variations in the number of viable probiotics between the 2% and 4% banana peel powder concentrations. These findings indicate that using a minimal quantity (2%) of banana peel powder can effectively serve as a prebiotic, promoting the growth of lactic acid bacteria. Moreover, Akter et al. [46] reported that the banana peel extract, after being dried, was found to have a total of 354.14 mg/g of reducing sugars, primarily composed of glucose and fructose. The utilisation of banana peel powder is prevalent in enhancing the functional attributes of probiotic yoghurt and milk supplements.

## 3. Materials and Methods

### 3.1. Banana Peel Preparation and Processing Flow Diagram

Fresh-picked bananas were harvested commercially in Chiang Mai in 2022. Hand-drawn qualitative process flow diagrams (PFDs) were used to document postharvest and handling steps sequentially, and mass streams flowed through banana pre-processing performed by a dried banana manufacturer in Chiang Mai, Thailand, similar to the study by Sunanta et al. [47]. After harvest, the samples were immediately transported to the factory for the same minimal processing procedure as the one performed by the dried banana manufacturer. The colour transition of banana peels during the storage was measured using a colourimeter (NR60CP precision colorimeter, Shenzhen ThreeNH Technology Co., Ltd., Shenzhen, China). In addition, the weight changes during the storage were weighed. It involved conducting three replicates, with each replicate consisting of three bunches of bananas. In total, six replicates were taken for each bunch.

During the peeling process, the ground was covered with canvas and the entire process was performed on the canvas. The significant residues, including banana bunch stalks and peels, were identified as by-products. Each of the residue was carefully weighed. The banana peels (BPs) were the main by-product from the processing. They were immediately transported to the laboratory and then dried at 50 °C in a hot-air oven for 48 h. The dried peels were then ground into a powder and sieved through a mesh (size 42 µm). Banana powder was kept at −4 °C for further analysis.

### 3.2. Proximate Analysis 

#### 3.2.1. Ash Determination

The inorganic residue that was left over after the organic materials were burned off is referred to as the ash content. The porcelain crucible was dried at 105 °C for 60 min, cooled in the desiccator, and then weighed. The sample was weighed and placed in a muffle furnace that was preheated to 550 °C for one night. The sample was taken out of the furnace after ashing. After cooling in a desiccator, the crucible was reweighed. The following equation was used to obtain the total ash content:

(1) $$\mathrm{Ash}\;(\%)=\frac{W2-\;W0}{W1}\times 100\%$$

where W0 = crucible weight; W1 = sample weight; and W2 = crucible and ash sample weight.

#### 3.2.2. Water Content Determination 

Fresh BPs (2.0 g) were added to a crucible that had already been dried and weighed. The materials were dried using oven combustion (00, 00) at 105 °C until a consistent weight was achieved. The samples were taken out of the oven, placed in a desiccator to cool for 60 min, and then weighed again. The water content was estimated as a percentage based on the weight lost from the original sample. Peels from bananas were cleaned and left to air-dry. Peels were dried at 50 °C and ground into a fine powder known as BP powder [48].

#### 3.2.3. Crude Protein Determination 

We performed crude protein determination using the same method used by Kjeldahl [49], in which crude protein (%) was computed using the following calculations:

(2) $$\mathrm{Nitrogen}\;\mathrm{in}\;\mathrm{sample}\;(\%)=\frac{W2\times \;\mathrm{Normality}\;\mathrm{of}\;\mathrm{acid}\;\times 14}{W1}\;\times 100\%$$

where W1 = sample weight measured by milligram and W2 = volume of HCl. 

The calculated total nitrogen was converted to protein using the following equation, where the conversion factor was 6.25:

Crude protein (%) = Nitrogen (%) × 6.25(3)

#### 3.2.4. Crude Lipid Determination

Exhaustive Soxhlet extraction was performed to identify the crude lipid content using petroleum ether. The sample was placed into pre-weighed thimbles with a total weight of 1.0 g and covered with a cotton wad. Each sample was placed in a Soxhlet extraction cup, weighed, and then into which 100 mL of petroleum ether was added. The solvent was then evaporated following the extraction. The extraction cup was taken out and put in a 105 °C oven to dry. After 60 min, the extraction cup was taken out of the oven and allowed to cool in desiccators. The crude lipid content was calculated using the following method after the lipid was measured by reweighing the extraction cup:

(4) $$\mathrm{Crude}\;\mathrm{lipid}\;(\%)=\frac{W3-\;W1}{W2}\times 100\%$$

where W1 = container weight; W2 = sample weight; and W3 = lipid and container weight. 

#### 3.2.5. Carbohydrate Determination

Nitrogen-free extract (carbohydrate) was calculated using the equation below:

NFE % = [100 − (WC % + CP % + CL % + CF % + ash %)(5)

where NFE = nitrogen-free extract; 

WC = water content; 

CP = crude protein; 

CL = crude lipid; 

CF = crude fibre. 

#### 3.2.6. Crude Fibre Determination

A clean, previously weighed filter crucible was used to weigh three identical, fat-free dry samples weighing 1.0 g [50]. The sample was placed in the crucible and moved to the hot extraction unit where it was digested for 30 min with 150 mL of a solution made up of 12.5% sulfuric acid and octanol. After 30 min, the condenser was turned off and left to cool. By suction, the acid solution was filtered and thoroughly cleaned with hot distilled water. To dissolve the alkali-soluble material from the sample, 150 mL of an alkali solution (12.5% sodium hydroxide) and 0.25 mL of octanol were added to the sample and digested for 30 min. The final residue and porcelain crucibles were dried at 105 °C in an oven for 60 min, cooled in a desiccator, and weighed after. The residue was ashed for 180 min at 550 °C in a muffle furnace that had been preheated. After the flask was once again cooled, the ultimate weight of the crucible and the ash was recorded, along with the weight differential. The following equation was used to compute the percentage of crude fibre content:

(6) $$\mathrm{Crude}\;\mathrm{fibre}\;(\%)=\frac{W2-\;W3}{W1}\times 100\%$$

where W1 = sample weight; 

W2 = crucible with residue weight; 

W3 = empty crucible with ash residue weight. 

### 3.3. Sequentially Solvent Extractions and Glycoarray Composition of Banana Peel

BP (100 g) was serially macerated with dichloromethane to remove fat soluble components, followed by ethanol to obtain ethanolic soluble fraction (ESF) [51,52]. The crude ESF was obtained by evaporating off the methanol to dryness and used for further characterisation and functional properties’ evaluation. The alcohol insoluble fraction (AIF) was the remaining insoluble residue after these sequentially solvent extractions. 

Glycan compositions of AIF were determined using microarray polymer profiling (MAPP). Glycoarrays were prepared using the method described by CR Bakshani, F Cuskin, NJ Lant, HCL Yau, WGT Willats, and J Grant Burgess [53]. To extract the AIF, 10 mg of BPP was mixed with 30 µL/mg of 50 mM cyclohexan ediaminetetraacetic acid (CDTA) at pH 7.5. The mixture was then subjected to tissue lysis at a frequency of 27 S^−1^ for 2 min, followed by 10 S^−1^ for 2 h. After centrifugation at 15,000× *g* for 10 min, the resulting supernatant was collected and stored in clean Eppendorf tubes at 4 °C on a rotating shaker. For the second extraction, 30 µL/mg of 4 M NaOH with 0.1% (*w*/*v*) NaBH_4_ was added to the remaining pellet. The mixture was shaken and centrifuged as described above, and the resulting supernatant was stored. The third extraction involved adding 30 µL/mg of cellulase (*Bacillus* spp. 5A, NZYTech, Lisbon, Portugal) to the remaining pellet, along with 2 µL/mL in 20 mM Tris buffer at pH 8.8. The samples were incubated at 45 °C for 16 h in a shaking heat block (SciQuip). After centrifugation, the supernatant was recovered and stored as described above. All the extracted glycan fractions, including the defined glycan standards at a concentration of 1 mg/mL, were loaded into the wells of a 384-well microtiter plate (Greiner Bio-One, Frickenhausen, Germany). The samples were mixed with a printing buffer containing 47% glycerol, 52.9% dH_2_O, 0.06% Triton X-100, and 0.04% ProClin™ 200, in a 1:1 (*v*/*v*) dilution. The final volume of the mixture was adjusted to 40 µL. Subsequently, the samples were printed onto a nitrocellulose membrane using a microarray printing robot (Marathon Argus, Arrayjet, Roslin, UK). The printed glycoarrays were then subjected to probing with monoclonal antibodies (mAbs) specific to plant cell wall components as described in Table 5, which were obtained from PlantProbes (Newcastle, UK) and INRA (Paris, France). Following the probing step, the arrays were incubated with alkaline phosphatase-conjugated anti-rat or anti-mouse secondary antibodies, depending on the specific antibody used. The binding of the mAbs was visualised using nitro-blue tetrazolium (NBT) and 5-bromo-4-chloro-3′-indolyphosphate p-toluidine (BCIP). The developed arrays were scanned using a Canon CanoScan 8800F scanner (Tokyo, Japan), and the intensity of antibody binding was quantified using microarray analysis software (Array-Pro Analyzer 6.3 software, Media Cybernetics, Rockville, MD, USA). Mean spot signal intensity values were generated for each sample, with the highest mean signal being assigned a value of 100. All other values were then normalised accordingly.

### 3.4. Chemical Characterisation of Banana Peel Ethanolic Soluble Fraction 

#### 3.4.1. Raw Material Preparation

The BPs (*Musa*, sub cv. ‘Nam Wa Mali-Ong’) were obtained from a sun-dried banana factory (Kamtara Co., Ltd., Muang, Thailand) located in Lamphun province, Thailand. The fruits selected for this study were harvested at the commercial stage, at which point the peels were collected. In order to completely remove any remaining water, peels initially underwent a cleaning process using flowing tap water. The BP were dried using a hot-air oven set at a temperature of 60 °C until they attained a stable weight. Subsequently, peels were pulverised to generate fine particles and strained through a screen with a mesh size of 42 µm. By following the method described by Pyar [61], with just a few modifications, the maceration method was employed in the extraction process. BP powder (20 g) was immersed in 200 millilitres of dichloromethane for 24 h. Subsequently, the mixture was filtered to separate the water, followed by a rotary evaporator to remove the solvent. To obtain a crude extract from dichloromethane, the filtrate was subjected to two further soakings. Upon completion of the extraction process using dichloromethane, the precipitate was extracted by immersing the crude extract in 200 mL of methanol for 24 h. Water and residual solvent were then removed by filtration and rotary evaporation, respectively. To obtain a crude extract from methanol. The filtrate was subjected to two further soakings.

#### 3.4.2. Total Phenolic Content 

Using gallic acid as the reference, the total polyphenol content of the BP sample was assessed using the procedure outlined by Sunanta [47]. Briefly, 120 µL of 7.5% *w*/*v* NaCO_3_ solution was added after 30 µL of BP extract had been mixed with 150 µL of Folin–Ciocalteu reagent. The reaction mixture was incubated at room temperature for 60 min in the dark. A spectrophotometer (SPECTROstar Nano BMG lab-04 TECH, Ortenberg, Germany) was used to measure the absorbance at 765 nm, and the total amount of polyphenols was represented as mg of gallic acid equivalents (GAE)/g of the dried banana peel sample.

#### 3.4.3. Total Flavonoid Content 

BP’s total flavonoid content was examined using (+)-catechin as the reference point [47]. The BP extract (25 µL) was combined with 125 µL of distilled water, 7.5 µL of a 5% NaNO_2_ solution, and left to sit at room temperature for 5 min. Then, 15 µL of a 10% AlCl_3_·6H_2_O solution was introduced. A total volume of 50 µL of a 1 M NaOH solution and 27.5 µL of distilled water were added after 6 min of incubation. At 510 nm, the absorbance was measured using a spectrophotometer. The amount of total flavonoids in the ESF was calculated as mg (+)-catechin equivalents (CE)/g of dry matter.

#### 3.4.4. Detection of Polyphenols using Reverse-phase High-Performance Liquid 

In accordance with Tongkaew et al. [39], RP-HPLC-DAD was carried out using a modified gradient elution program. A volume of 1000 µL of a solution containing 50% ethanol was utilised to add moisture back to the dehydrated sample. Each sample was injected with a volume of 10 µL after being filtered via 0.45 µm PTFE membrane filters. The HPLC analysis was conducted using an Agilent 1100 series instrument, which consisted of a binary pump, a diode array detector, an autosampler, and a column compartment maintained at a temperature of 25 °C. The compounds (+)-catechin, gallic acid, p-coumaric acid, and rosmarinic acid were isolated using a C18 column (Poroshell 120 EC-C18 4.6 × 100 mm, 2.7 2.5 µm) and detected using a diode array detector (DAD) at a wavelength of 280 nm. The following elution gradients were used to separate phenolic compounds using mobile phase A (DI water and 0.1% formic acid) and B (acetonitrile and 0.1% formic acid): At the running time of 0–10 min the gradient of B increases from 5–15%; 10–20 min held at 15%; 20–30 min, 15–35%; 30–35 min, 35–100%; 35–40 min, 100% follow by de-creased the gradient of B from 100–5% at 40–41 min then held for 5 min, at a flow rate of 0.5 mL/min. All peaks’ spectra were collected between 190 and 400 nm, and they were compared to real standards of (+)-catechin, gallic acid, p-coumaric acid, and rosmarinic acid.

#### 3.4.5. ABTS Scavenging Activity

The assay for 2,2-azino-bis-(3-ethylbenzothiazoline-6-sulfonic acid)] was conducted following the method describe previously [47]. In brief, the two stock solutions (2.45 mmol/L of potassium persulfate solution and a 7.0 mmol/L of ABTS solution) were combined to create the working solution. The mixture of the solutions was performed in equal parts. The solution was diluted with methanol to achieve an absorbance of 0.7 ± 0.02 units at 734 nm after being left to react for 12–16 h at room temperature in the dark. After adding 10 µL of BP extract to the ABTS solution, mixing it, and letting it stand for 30 min, the absorbance at 734 nm was determined. The Trolox equivalent (TE) was measured in mg of dry mass.

#### 3.4.6. DPPH Scavenging Activity 

The free radical scavenging activity was determined by assessing the scavenging activity of the stable DPPH (2,2-diphenyl-1-picrylhydrazyl) free radical, and the calculation of this activity was based on previous research [47]. A solution of ethanol containing 0.2 mmol/L DPPH was combined with a sample, with a volume of 25 µL. The absorbance at 550 nm was measured using a spectrophotometer after incubating for 30 min at room temperature in the absence of light. The free radical scavenging activity is determined by measuring the extent of DPPH decolourisation.

#### 3.4.7. Ferric-Reducing Antioxidant Power (FRAP) 

The colorimetric approach, as reported by Liaqat et al. [62], was used to calculate FRAP. The FRAP reagent was made and combined (ratio 10:1:1) using Reagent A, which is 300 mM acetate buffer (pH 3.6), Reagent B, which is 10 mM TPTZ in 40 mM HCl, and Reagent C, which is 20 mM FeCl_3_.6H_2_O. After mixing 20 µL of ESF with 150 µL of FRAP solution, the mixture was left to sit in the dark for 30 min. The mixture’s absorbance was measured at 593 nm with a spectrophotometer. The utilised standard was ascorbic acid, and the FRAP was expressed as milligrams of ascorbic acid equivalent per milligram of dry mass.

### 3.5. Prebiotic Properties of Banana Peel Ethanolic Soluble Fraction

#### 3.5.1. Microorganisms

Four different kinds of microorganisms, consisting of two beneficial types, specifically *Lacticaseibacillus paracasei* (formerly *Lactobacillus paracasei*) [the Ministry of public health, Thailand (2011)] [43] and *Bifidobacterium longum* ATCCBAA-999, and two harmful types, notably *Escherichia coli* ATCC25922 and *Clostridium perfringens* ATCC3626, were used in this experiment. These are utilised in investigations related to the human microbiome. All representative strains obtained were provided by Innovation Centre for Holistic Health, Nutraceuticals and Cosmeceuticals, Faculty of Pharmacy, Chiang Mai University, Chiang Mai, Thailand. 

#### 3.5.2. Prebiotic Properties of Banana Peel Extract

Various carbon sources were tested in modified MRS broth containing 2% glucose (standard MRS broth), 2% of the ESF, and 2% (*w*/*v*) commercial inulin. The probiotic *L. paracasei* was cultured in standard MRS broth medium and *B. longum* was cultured in standard BSM medium for a duration of 72 h and were capable of being centrifuged at 10,000× *g* and 4 °C for 10 min while retaining only the cells. A microbial concentration of 10^6^ CFU/mL was achieved by diluting the bacteria in phosphate-buffered saline. The probiotic cell suspension was subsequently added to both the normal formula (with added glucose) and the free formula. However, a substantial quantity of 2% (*w*/*v*) was replaced with the required mixture of test substances. After 48 h, the culture was developed. For the pour plate technique on MRS agar and BSM agar as culture media, probiotic growth was sampled at hours 0 and 48, respectively.

The prebiotic properties of ESF were reported as a prebiotic index (PI) according to a method previously described by of Jaiturong et al. [63] and Kaewarsar et al. [43,64] with some modifications. Briefly, the samples were tested on the growth promotion of representative bacteria in the digestive tract. *L. paracasei* (Lac) and *B. longum* (Bif) were used as the representative probiotic cultures, while *E. coli* (Eco) and *C. perfringens* (Clos) were used as the representative enteric species. Inulin, a commercial prebiotic, was used as the reference standard for comparison. Each assay was performed in triplicate, measuring the number of viable Log10 colony-forming units (Log_10_ CFU) per ml at T0 for 0 h and at T48 incubation for 48 h, respectively, on 2% *w*/*v* dextrose (standard carbon source in common medium as control), 2% *w*/*v* test banana peel extract, and 2% *w*/*v* inulin as the commercial prebiotic. The prebiotic index (PI) was computed using the following equation:

$$\mathrm{Prebiotic}\;\mathrm{Index}\;(\mathrm{PI})=\frac{\mathrm{Bif}\;T48-\mathrm{Bif}\;T0}{\mathrm{Total}\;T48-\mathrm{Total}\;T0}-\frac{\mathrm{Eco}\;T48-\mathrm{Eco}\;T0}{\mathrm{Total}\;T48-\mathrm{Total}\;T0}+\frac{\mathrm{Lac}\;T48-\mathrm{Lac}\;T0}{\mathrm{Total}\;T48-\mathrm{Total}\;T0}-\frac{\mathrm{Clos}\;T48-\;\mathrm{Clos}\;T0}{\mathrm{Total}\;T48-\mathrm{Total}\;T0}$$

where Bif = bifidobacterial numbers at sample time/number at inoculation; 

Lac = lactobacilli numbers at sample time/number at inoculation;

Eco = E. coli-Enterobacter group numbers at sample time/number at inoculation; 

Clos = clostridia numbers at sample time/number at inoculation;

Total = total bacteria numbers at sample time/numbers at inoculation.

### 3.6. Statistic Analysis

The experiments were carried out using a completely randomised design (CRD) with 18 for physical analyses and 6 replicates for chemical analyses. The results of the data are presented as the mean ± standard deviation (SD). The statistical analysis employed in this study involved the use of one-way analysis of variance (ANOVA). The Duncan’s multiple range test was used to access the difference between the means at a significance level of *p* < 0.05. The independent *t*-test with a two-tail *p* value was used to compared two values. The statistical analyses were conducted using SPSS version 24.0, a software developed by the SPSS Institute in Armonk, NY, USA.

## 4. Conclusions

This study examined the physical, chemical, and functional characteristics of banana peels in order to determine their potential usefulness as a valued resource. It revealed that the processing of dried bananas resulted in the production of banana peels, which accounted for more than 24% of the overall production and determined as by-product. The colour of banana peels undergoes a transition from green to yellow during storage as a consequence of chlorophyll degradation. After conducting the proximate analysis, the results found that carbohydrates were the primary constituents. Moreover, the analysis of glycoarray profiling also identified unique glycans present in banana peels, which were specifically recognised by certain monoclonal antibodies. Furthermore, extracts derived from banana peels contained phenolic and flavonoid compounds, as well as their antioxidant activity as measured by ABTS, DPPH, and FRAP assays. The HPLC study also detected different phenolic substances ((+)-catechin, gallic acid, p-coumaric acid, rosmarinic acid). Importantly, the extracts derived from banana peels exhibited a prebiotic impact by stimulating the proliferation of advantageous bacteria while inhibiting the growth of potential pathogens. Consequently, the prebiotic index scores demonstrated that banana peels had a greater capacity to promote the growth of beneficial bacteria in comparison to regular sugar. The results pointed out that banana peels have the potential to be a useful source of dietary fibre, bioactive chemicals, and prebiotic elements. Additional investigation is strongly recommended to delve into the enhancement of extraction procedures and the advancement of applications in functional foods and nutraceuticals.

## Figures and Tables

**Table 1:** The colour and weight changes during the storage of fresh-picked bananas.

Day of Ripening	L*	a*	b*	$\Delta E^{*}$	Weight Loss (%)
Day 0	32.01 ± 1.37 ^a^	−8.78 ± 0.22 ^a^	54.81 ± 2.31 ^ab^	0.00 ± 0.00 ^a^	0.00 ± 0.00 ^a^
Day 1	29.75 ± 1.64 ^a^	−5.39 ± 1.08 ^ab^	48.89 ± 2.29 ^a^	9.74 ± 1.26 ^b^	2.18 ± 0.15 ^ab^
Day 2	30.33 ± 1.15 ^a^	−1.11 ± 0.81 ^bc^	51.86 ± 1.94 ^a^	12.31 ± 1.01 ^b^	4.15 ± 0.44 ^bc^
Day 3	33.11 ± 1.31 ^ab^	−0.47 ± 0.85 ^bcd^	55.18 ± 1.99 ^ab^	12.20 ± 2.20 ^b^	6.57 ± 0.87 ^cd^
Day 4	31.74 ± 1.24 ^a^	0.04 ± 1.02 ^cd^	52.26 ± 1.75 ^a^	10.82 ± 1.88 ^b^	8.64 ±1.20 ^de^
Day 5	35.74 ± 1.48 ^ab^	0.87 ± 1.00 ^cd^	60.55 ± 2.93 ^b^	14.84 ± 2.13 ^bc^	10.46 ± 1.39 ^ef^
Day 6	34.83 ± 2.13 ^ab^	4.55 ± 1.06 ^de^	53.79 ± 2.99 ^ab^	15.38 ± 2.26 ^bc^	12.20 ± 1.46 ^fg^
Day 7	33.84 ± 2.17 ^ab^	6.72 ± 1.34 ^e^	50.77 ± 2.79 ^a^	18.94 ± 3.43 ^cd^	13.62 ± 1.52 ^fg^
Day 8	38.84 ± 1.79 ^b^	12.53 ± 1.45 ^f^	54.55 ± 2.16 ^ab^	23.67 ± 1.06 ^de^	15.27 ± 1.49 ^gh^
Day 9	32.30 ± 2.14 ^a^	13.84 ± 1.31 ^f^	48.34 ± 2.80 ^a^	26.29 ± 2.23 ^e^	17.89 ± 1.36 ^h^

Values are expressed as mean with SD. The different letters in the same column indicate statistically significant differences at *p* < 0.05.

**Table 2:** Proximate analysis of banana peels.

Proximate Analysis	Percentage
Ash	8.20 ± 0.15
Moisture	4.93 ± 0.04
Protein	3.76 ± 0.12
Lipid	5.22 ± 0.11
Carbohydrate	61.34 ± 0.37
Fibre	16.56 ± 0.04
Reducing sugar	0.45 ± 0.27
Total sugar	0.21 ± 0.02

Values are expressed as mean with SE.

**Table 3:** Chemical properties of banana peel extract.

Chemical Properties	Methanol	Dichloromethane	*p*
Total phenolic content (mg gallic/g dry weight)	30.71 ± 3.15	36.23 ± 1.87	0.222
Total flavonoid content (mg (+)-catechin/g dry weight)	12.82 ± 2.05	27.99 ± 2.01	0.006
(+)-Catechin (mg/g dry weight)	0.24	ND	-
Gallic acid (mg/g dry weight)	0.22	ND	-
p-Coumaric acid (mg/g dry weight)	ND	0.58	-
Rosmarinic acid (mg/g dry weight)	ND	0.93	-
ABTS (mg trolox/g dry weight)	34.36 ± 6.22	15.31 ± 0.43	0.091
DPPH (mg trolox/g dry weight)	52.38 ± 2.04	42.66 ± 1.75	0.022
FRAP (mg ascorbic acid/g dry weight)	0.36 ± 0.01	0.15 ± 0.01	0.000

Values are expressed as mean with SE. ND means not detected. The differential test is the statistical analysis using independent *t*-test with a 2-tail *p* value.

**Table 4:** Prebiotic index (PI) score of bacteria in MRS agar with glucose (control) and prebiotics.

Carbon Source	Prebiotic Index
Glucose	0.43 ± 0.07 ^b^
Banana peel	1.64 ± 0.09 ^a^
Inulin	1.77 ± 0.07 ^a^

Value was expressed as mean with SE. The different letters in the same column indicate statistically significant differences at *p* < 0.05.

**Table 5:** Specificity of the monoclonal antibodies.

Probe	Specificity	References
Arabinogalactan protein glycan		
LM2	Arabinogalactan protein glycan, β-linked GlcA	WG Willats and JP Knox [54], S Pattathil, U Avci, D Baldwin, AG Swennes, JA McGill, Z Popper, T Bootten, A Albert, RH Davis, C Chennareddy et al. [33]
JIM13	Arabinogalactan protein glycan	WG Willats and JP Knox [32]
Rhamnogalacturonan-I		
LM5	β(1,4)-Galactan	P Sutherland, I Hallett and M Jones [34], WG Willats and JP Knox [54], S Pattathil, U Avci, D Baldwin, AG Swennes, JA McGill, Z Popper, T Bootten, A Albert, RH Davis, C Chennareddy et al. [33]
LM6	(1→5)-α-L-Arabinan	P Sutherland, I Hallett and M Jones [34], WG Willats and JP Knox [54]
LM6-M	(1→5)-α-L-Arabinan	V Cornuault, F Buffetto, SE Marcus, M-J Crépeau, F Guillon, M-C Ralet and JP Knox [55]
LM16	Processed (1→5)-α-L-arabinan	MG Rydahl, AR Hansen, SK Kračun and J Mravec [56]
Homogalacturonan		
LM19	Homogalacturonan, unesterified	P Sutherland, I Hallett and M Jones [34]
LM20	Homogalacturonan	P Sutherland, I Hallett and M Jones [34]
JIM7	Partially me-homogalacturonan (non-blockwise) pectin	S Pattathil, U Avci, D Baldwin, AG Swennes, JA McGill, Z Popper, T Bootten, A Albert, RH Davis, C Chennareddy et al. [33]
Heteroxylan		
LM10	(1,4)-β-D-Xylan	P Sutherland, I Hallett and M Jones [34], S Pattathil, U Avci, D Baldwin, AG Swennes, JA McGill, Z Popper, T Bootten, A Albert, RH Davis, C Chennareddy et al. [33], MG Rydahl, AR Hansen, SK Kračun and J Mravec [56]
LM11	(1,4)-β-D-Xylan/arabinoxylan	P Sutherland, I Hallett and M Jones [34], S Pattathil, U Avci, D Baldwin, AG Swennes, JA McGill, Z Popper, T Bootten, A Albert, RH Davis, C Chennareddy et al. [33], SE Marcus, AW Blake, TAS Benians, KJD Lee, C Poyser, L Donaldson, O Leroux, A Rogowski, HL Petersen, A Boraston et al. [57]
LM28	Glucuronoxylan	V Cornuault, F Buffetto, MG Rydahl, SE Marcus, TA Torode, J Xue, M-J Crépeau, N Faria-Blanc, WG Willats and P Dupree [58]
Xyloglucan		
LM15	Xyloglucan, XXXG motif	P Sutherland, I Hallett and M Jones [34], S Pattathil, U Avci, D Baldwin, AG Swennes, JA McGill, Z Popper, T Bootten, A Albert, RH Davis, C Chennareddy et al. [33]
LM24	Galactosylated xyloglucan	MG Rydahl, AR Hansen, SK Kračun and J Mravec [56]
LM25	Galactosylated xyloglucan, XXXG motif	MG Rydahl, AR Hansen, SK Kračun and J Mravec [56]
LM21	Heteromannan	MG Rydahl, AR Hansen, SK Kračun and J Mravec [56], SE Marcus, AW Blake, TAS Benians, KJD Lee, C Poyser, L Donaldson, O Leroux, A Rogowski, HL Petersen, A Boraston et al. [57]
LM22	(1→4)-β-d-(Gluco)mannan	SE Marcus, AW Blake, TAS Benians, KJD Lee, C Poyser, L Donaldson, O Leroux, A Rogowski, HL Petersen, A Boraston et al. [57]
INCh-1	α-(1-4)-Glucan (starch)	MG Rydahl, AR Hansen, SK Kračun and J Mravec [56]
Extensin		
LM1	Extensin	M Smallwood, H Martin and JP Knox [59]
JIM20	Extensin	M Smallwood, A Beven, N Donovan, S Neill, J Peart, K Roberts and J Knox [60]
